# Identification of cotton *MOTHER OF FT AND TFL1* homologs, *GhMFT1* and *GhMFT2*, involved in seed germination

**DOI:** 10.1371/journal.pone.0215771

**Published:** 2019-04-19

**Authors:** Xiuli Yu, Hui Liu, Na Sang, Yunfei Li, Tingting Zhang, Jie Sun, Xianzhong Huang

**Affiliations:** 1 The Key Laboratory of Oasis Eco-Agriculture, College of Agriculture, Shihezi University, Shihezi, Xinjiang, China; 2 Special Plant Genomics Laboratory, College of Life Sciences, Shihezi University, Shihezi, Xinjiang, China; Texas Tech University, UNITED STATES

## Abstract

Plant phosphatidylethanolamine-binding protein (PEBP) is comprised of three clades: FLOWERING LOCUS T (FT), TERMINAL FLOWER1 (TFL1) and MOTHER OF FT AND TFL1 (MFT). FT/TFL1-like clades regulate identities of the determinate and indeterminate meristems, and ultimately affect flowering time and plant architecture. MFT is generally considered to be the ancestor of FT/TFL1, but its function is not well understood. Here, two *MFT* homoeologous gene pairs in *Gossypium hirsutum*, *GhMFT1-A/D* and *GhMFT2-A/D*, were identified by genome-wide identification of *MFT*-like genes. Detailed expression analysis revealed that *GhMFT1* and *GhMFT2* homoeologous genes were predominately expressed in ovules, and their expression increased remarkably during ovule development but decreased quickly during seed germination. Expressions of *GhMFT1* and *GhMFT2* homoeologous genes in germinating seeds were upregulated in response to abscisic acid (ABA), and their expressions also responded to gibberellin (GA). In addition, ectopic overexpression of *GhMFT1* and *GhMFT2* in *Arabidopsis* inhibited seed germination at the early stage. Gene transcription analysis showed that ABA metabolism genes *ABA-INSENSITIVE3* (*ABI3*) and *ABI5*, GA signal transduction pathway genes *REPRESSOR OF ga1-3* (*RGA*) and *RGA-LIKE2* (*RGL2*) were all upregulated in the *35S*:*GhMFT1* and *35S*:*GhMFT2* transgenic *Arabidopsis* seeds. GhMFT1 and GhMFT2 localize in the cytoplasm and nucleus, and both interact with a cotton bZIP transcription factor GhFD, suggesting that both of GhMFT1, 2 have similar intracellular regulation mechanisms. Taken together, the results suggest that *GhMFT1* and *GhMFT2* may act redundantly and differentially in the regulation of seed germination.

## Introduction

In the life cycle of higher plants, the transition from vegetative to reproductive growth (floral transition) is controlled by endogenous cues and external cues [1–3]. Previous studies on the molecular mechanisms of flowering initiation in *Arabidopsis thaliana* have uncovered six major pathways to orchestrate flowering time, such as photoperiod, vernalization, ambient temperature, gibberellin (GA), autonomous and age pathways [4]. Another transition in a flowering plant is from embryonic growth to germination growth [5]. Seed dormancy can form a soil seed bank and open a protective mechanism for the seed in an unfavorable environment [6]. Seed germination plays an essential part during the growth of the descendants and it is a prerequisite for crop yield [7]. Breaking dormancy is affected by internal cues and external cues [8], which are primarily controlled by two major antagonistic phytohormones, abscisic acid (ABA) and GA [9]. It is well known that ABA functions in developmental processes of many plants like seed germination and dormancy. ABA inhibits seed germination by hampering water absorption as well as the rupture of seed coat and endosperm [10, 11]. The extensive molecular genetics analyses reveal that ABA-INSENSITIVE3 (ABI3) and ABI5 are the essential regulators in regulating ABA to boost seed dormancy and suppress seed germination [12–15]. GA can counteract the effects of ABA and promote seed germination, plant growth and early flowering. During seed germination, GA accumulation is accompanied by a decrease of ABA content, indicating that GA and ABA play opposite roles in seed germination [16, 17]. GA3-oxidase (GA3OX) is a pivotal enzyme controlling GA biosynthesis, which is encoded by four genes *GA3OX1*, *GA3OX*2, *GA3OX3* and *GA3OX4*. The *ga3ox1 ga3ox2* double mutant exhibits serious defects in seed germination, indicating that *GA3OX1* and *GA3OX2* are crucial genes regulating seed germination [18, 19]. DELLA proteins, consisting of REPRESSOR OF ga1-3 (RGA), GA-INSENSITIVE (GAI), and RGA-LIKE1-3 (RGL1-3) [20–24], act as repressors in regulating GA signaling, among which RGA and RGL2 play roles in inhibiting seed germination by mediating the interaction between GA and ABA [14, 22, 25].

*MOTHER OF FT AND TFL1* (*MFT*), which encodes a phosphatidylethanolamine-binding protein (PEBP), has an evolutionary and functionally conserved role in most plants [26–29]. In *A*. *thaliana*, the PEBP family genes are mainly composed of three clades: *FT*-like, *TFL1*-like and *MFT*-like [30]. It is generally considered that *MFT*-like branch is the evolutionary ancestor of the other two branches [26, 29]. *FT*-like and *TFL1*-like are two important regulators with opposite functions in the control of flowering time and plant architecture [31–36]. Compared with *FT* and *TFL1*, the exact biological function of *MFT* is not well understood. There is only one *MFT* gene in *Arabidopsis* genome [37]. Overexpression of *MFT* leads to slightly early flowering compared with that of wild-type plants, and loss-of-function mutation in *MFT* does not exhibit observable phenotypes, suggesting that *MFT* acts redundantly in regulating flowering time in *Arabidopsis* [37]. *MFT* homologs have been characterized from several plant species, and recent studies have shown that they have different roles in controlling flowering time in respective species. For example, Hou et al. [38] reported that *Adiantum capillus-veneris MFT* (*AcMFT*) accelerated the fioral transition and partially rescued the late flowering phenotype of *Arabidopsis ft-1* mutant. However, two groups reported that both *Dendrobium nobile MFT* (*DnMFT*) [39] and *Hevea brasiliensis MFT1* (*HbMFT1*) [40] delayed flowering time. Several studies have reported that *MFT* homologs do not affect the flowering transition: *Populus nigra* [41], *Populus* [42], *Picea abies* [27], *Symplocarpus renifolius* [43], *Glycine max* [44], *Actinidia chinensis* [45] and *Citrus latifolia* [46]. In addition to regulating flowering, *MFT* homologs have also been found to be involved in seed dormancy and germination [5, 40, 44, 47–53]. In *Arabidopsis*, *MFT* functions as a negative regulator of germination under far-red light conditions by modulating ABA and GA signaling [50]. Expression of *MFT* is directly regulated by two key transcription factors, ABI3 and ABI5, in response to ABA [5]. *Triticum aestivum MFT* (*TaMFT*) also inhibits seed germination and functions as a positive regulator of dormancy [47].

The allotetraploid cotton (*Gossypium hirsutum* and *Gossypium barbadense*) with a complex genome is the world’s leading sources of natural fiber crops and crude oil [54]. In recent years, the whole genome has been sequenced successfully in cotton (*Gossypium* spp.), which provides a resource for characterization of gene family [55–62]. Genome-wide analysis reveals that there are at least eight PEBP homoeologous gene pairs in tetraploid *G*. *hirsutum* [63–65]. Our group has demonstrated that the *FT* homolog *GhFT1* controls flowering time and *TFL1* homolog *SELFING-PRUNING* (*GhSP*) regulates fruiting branches architecture in cotton [66–68], and similar findings have also been reported in other groups [69–71]. However, the exact biological functions of *GhMFT* homologs in Upland cotton remain unclear. We characterized two *MFT* homoeologous genes, *GhMFT1* and *GhMFT2*, from *G*. *hirsutum* in this study. We found that their expressions were upregulated significantly in the developing ovule and their expressions were obviously declined and responded to ABA and GA during seed germination. Furthermore, ectopic overexpression of *GhMFT1 and GhMFT2* in *Arabidopsis* inhibited seed germination at the early stage. These preliminary results suggest that *GhMFT* homologs may be involved in ovule development and serve as the potential negative regulators in seed germination.

## Materials and methods

### Plant materials and growth conditions

The seeds of *G*. *hirsutum* cv. Xinluzao 33 were planted in the experimental fields of Shihezi University (Xinjiang, China). *A*. *thaliana mft-2* (in the wild type (Columbia, Col-0) background) was ordered from the Arabidopsis Biology Resources Center (ABRC, Columbus, OH, USA). Seeds of wild type and *mft-2* were surface sterilized and planted as described previously [66]. After 10 days, the seedlings were transplanted to soil in a growth chamber under long-day (LD) conditions (16-h-light/8-h-dark, ambient temperature of 22°C, light intensity of 200 μmol photons m^–2^s^–1^). For tissue expression analysis, roots, stems, shoot apical meristems (SAM) and leaves were collected at the third true leaf expanding stage (20 days after planting). A whole flower was collected at the flowering stage. Cotton bolls were harvested at the following time-points during development: –3 and 0 d of anthesis (DOA) ovules, 3 and 8 d of post-anthesis (DPA) ovules which contain initiating fiber cell, and 12–30 of DPA ovules. All samples collected were immediately frozen in liquid nitrogen and stored at –80°C.

### Sequence alignment and phylogenetic analysis

Cotton *MFT* homologous genes were obtained through tBLSATn searches using AtMFT as query against the Upland cotton (AD)_1_ tetraploid genome of *G*. *hirsutum* [58, 60], the sea land cotton (AD)_2_ tetraploid genome of *G*. *barbadense* [59], the A_2_ genome of diploid *G*. *arboreum* [57] and the D_5_ genome of diploid *G*. *raimondii* [56] at COTTONGEN (http://www.cottongen.org; S1 Table). The amino acid sequences of MFT proteins from other plant species used in this study were downloaded from NCBI non-redundant database (S2 Table). Multiple sequence alignment was performed with Clustal W using the default parameters. A phylogenetic tree was constructed using Molecular Evolutionary Genetics Analysis software 6.0 [72] (neighbor-joining, Poisson correction distance model). The nodal reliability in the tree was evaluated by bootstrap analysis with 1000 replicates. Putative cis-acting regulatory elements of *MFT* promoters were analyzed based on the description of Xi et al. [5].

### Gene expression analysis

Total RNA for each sample was isolated using the RNAprep Pure Plant Kit (Polysaccharides & Polyphenolics-rich) (TIANGEN, Beijing, China) according to the manufacturer’s protocol. Total RNA was reversed to cDNA using an M-MLV Reverse Transcriptase Kit (Bioteke Corporation, Beijing, China). Quantitative Real-time PCR (qRT-PCR) was carried out on an Applied Biosystems 7500 Fast Real-Time PCR System (Life Technologies, Carlsbad, CA, USA) in a 20 μL volume containing 100 ng of cDNA, 4 pM of each primer, and 10 μL SYBR Green PCR Master Mix system (TaKaRa). The PCR conditions and the calculation method of gene expression were the same as what had been described previously [68]. Information on the qRT-PCR primers for gene expression analysis and gene cloning used in this study was listed in S3 Table. The nucleotide sequences of *GhMFT* homoeologous genes marked with primer location for qRT-PCR were shown in S1 Fig. A cotton *Ubiquitin7* (*GhUBQ7*, GenBank accession no. DQ116441) gene and an *Arabidopsis Actin2* (AT3G18780) gene were used as internal controls, respectively. Three replicate assays were conducted with separately isolated RNA, and three technical triplicates were performed for each PCR reaction.

### Plasmids construction and *Arabidopsis* transformation

Complete open reading frame (ORF) cDNAs of *GhMFT1* and *GhMFT2* were amplified by RT-PCR using gene specific primers (S3 Table). The ORF cDNAs were separately cloned into *pMD*^*TM*^*19-T* Vector Cloning Kit (TaKaRa). Two constructs confirmed by sequences analysis were then introduced into *pCAMBIA2300-35S-OCS* [66] binary vectors containing downstream of the cauliflower virus 35S promoter to generate *35S*:*GhMFT1* and *35S*:*GhMFT2*. *Agrobacterium tumefacien* strains GV3101, harboring *35S*:*GhMFT1* and *35S*:*GhMFT2* constructs, were used to be transformed into wild type and *mft-2* mutant plants using the floral dip method [73], respectively. Homozygous transgenic plants were screened and identified as described by Guo et al. [66]. Flowering time was monitored as the number of rosette leaves per plant and days to flowers for the first flower bloomed after transplanting the seedlings into the soil [68].

### Seed germination assays

100 mature cotton seeds with uniform size were sterilized with 0.1% (w/v) mercuric chloride for 10 min, and then rinsed several times in sterile water. After removing the seed coats, the sterilized seeds were sown on the Petri dishes with half-strength Murashige and Skoog (MS) salt (Murashige & Skoog, Duchefa, pH 5.7) mixture, 1% (w/v) sucrose and 0.8% (m/v) agar, and the number of germinating seeds was counted. For the abiotic treatments, the sterilized seeds were planted on half-strength MS medium supplemented with 50 μM GA_3_ (Sigma-Aldrich) and 100 μM ABA (Sigma-Aldrich) according to the previous studies [74, 75], respectively. Petri dishes were placed in a phytotron at 28°C under dark conditions. When the primary root length reaches 1 mm, the seed is considered to be germinated [76]. Seeds were collected at 12 h and 24 h of imbibition for RNA isolation.

Seeds of wild type, *mft-2* and each transgenic homozygote were sterilized as described previously [66]. Sterilized seeds were then plated on the Petri dishes with half-strength MS medium. Petri dishes were placed in a phytotron at 22°C under LD conditions (16-h-light/8-h-dark). The method of abiotic stress treatments was as described above. The concentrations of GA_3_ and ABA are 5 μM and 10 μM, respectively. For the germination assay, at least 100 seeds for each genotype were observed, and the germination percentage was calculated according to three independent experiments.

### Subcellular localization analysis

To analyze the subcellular localization of the GhMFT1 and GhMFT2 proteins, we utilized the *35S*:*GFP* vector constructed by Guo et al. [66]. The coding regions of *GhMFT1* and *GhMFT2* without stop codon were separately amplified by PCR and inserted into the *Kpn* I and *Bam*H I sites of the *35S*:*GFP* vector to generate *35S*:*GhMFT1-GFP* and *35S*:*GhMFT2-GFP* in-frame fusions which were transformed into *A*. *tumefaciens* strains GV3101, respectively. The abaxial surface of leaf blade of *Nicotiana benthamiana* was then infiltrated with *A*. *tumefaciens* strains according to the description of Si et al. [68]. The infiltrated leaves were used to detect GFP fluorescence using a confocal laser scanning microscopy (CLSM510; Zeiss, Jena, Germany).

### Yeast two-hybrid assays

The coding sequences of *GhMFT1* and *GhMFT*2 were amplified and cloned into *pGBKT7* (Clontech) to produce *BD-GhMFT1* and *BD-GhMFT2*, respectively. *AD-GhFD* was constructed by Si et al. [68]. Yeast two-hybrid assays were carried out according to the method of Si et al. [68].

### Bimolecular fluorescence complementation (BiFC) assays

The coding regions of *GhMFT1* and *GhMFT2* were separately amplified and cloned into the *pDONRZeo* vector (Invitrogen) for fusion with the N-terminus of PVYNE [77] by LR reaction. *GhFD* coding region has been amplified and fused into the C-terminus of *PSCYCE* vectors [68]. The BiFC assays were performed as described by Si et al. [68].

## Results

### Identification and phylogenetic analysis of *GhMFT* homologs from *G*. *hirsutum*

**Fig 1:**
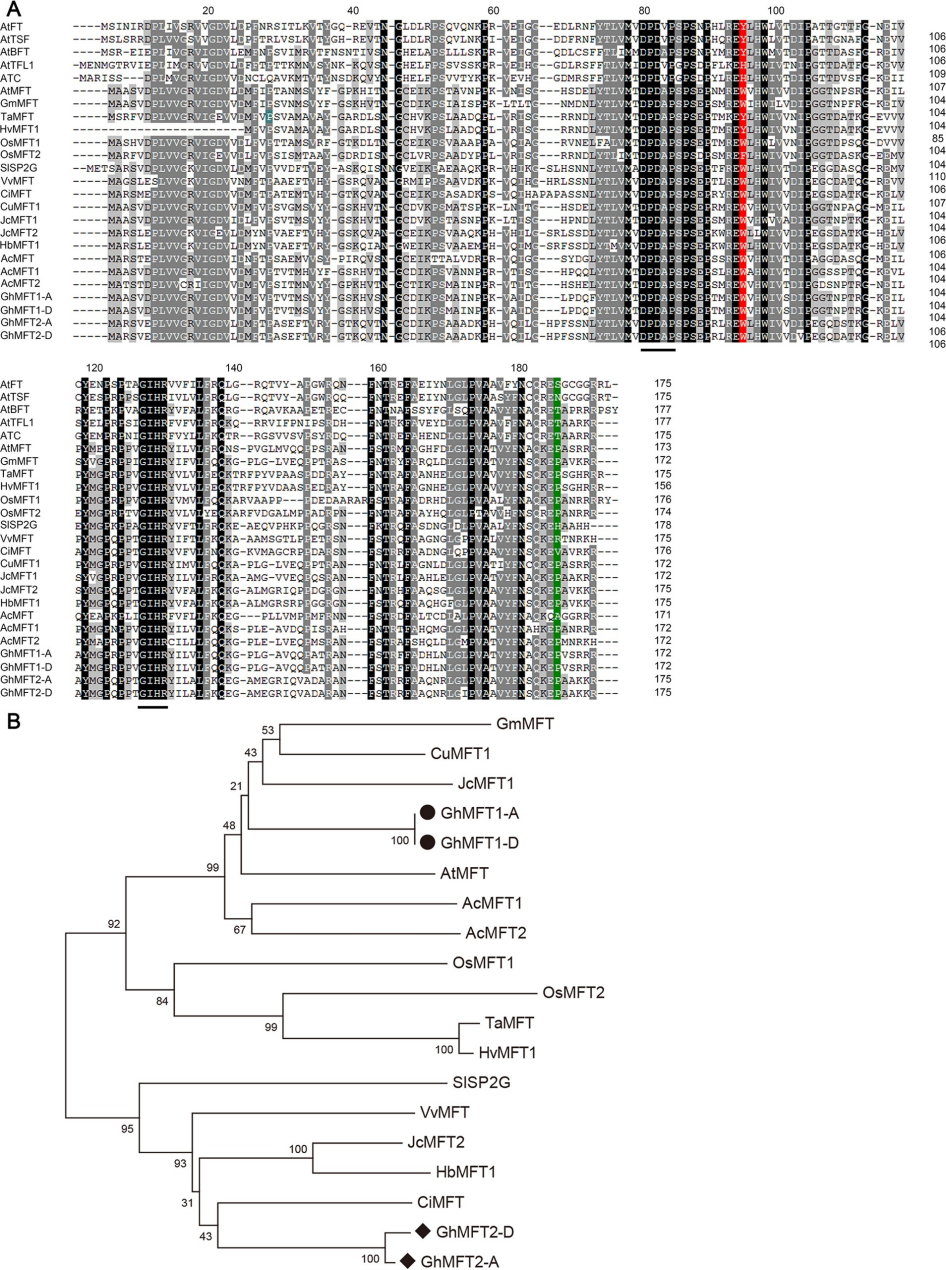
Bioinformatics analysis of amino acid sequences of plant PEBP family. (A) Multiple alignment of amino acid sequences of plant PEBP family. Black letters indicate the identical amino acids. Black lines indicate conserved D-P-D-x-P and G-x-H-R motifs of PEBP proteins. Amino acids shown in red and green indicate three conserved amino acid His (H)/Tyr (Y)/Trp (W) residues for FT/TFL1/MFT-clade and Pro (P) residue for MFT-clade, respectively. (B) Phylogenetic analysis of plant PEBP family based on amino acid sequences. The bootstrap consensus tree was inferred from 1000 replicates using MEGA 6.0 [72].

Genome-wide identification revealed that there were two *MFT* loci in each A_2_ and D_5_ diploid cotton genome and four in each tetraploid genome with two homoeologous genes in each A and D subgenome of the (AD)_1_ and (AD)_2_ tetraploid (S1 Table). Our findings are in good agreement with those of published papers [64, 65, 71]. To explore their functions in Upland cotton, their ORF sequences were successfully cloned from *G*. *hirsutum* using gene specific primers (S3 Table). *GhMFT1* ORF is 519 bp encoding a protein of 172 aa; and *GhMFT2* ORF is 528 bp encoding a protein of 175 aa (Panel A in S2 Fig). Gene structure analysis revealed that *GoMFTs* contain four exons and three introns, which share genomic characteristic of plant PEBP family genes (Panel B in S2 Fig). Multiple amino acid alignment including GoMFTs and other plant PEBP homologs (S2 Table) indicated that GoMFT proteins contain the conserved D-P-D-x-P and G-x-H-R motifs (Fig 1A), which are also present in both FT-like and TFL1-like proteins. The conserved key amino acid residues Tyr85 (Y)/His88 (H) in FT/TFL1-like protein are replaced by Trp83/85 (W) in GoMFT1 and GoMFT2, respectively, suggesting that GoMFT homologs do not play a central role in flowering time control. There is a conserved proline (P) at the end of the carboxyl group, which is only found in MFT-like, but no similar amino acid has been found in FT/TFL1-like [26]. Phylogenetic analysis revealed that GhMFT1 showed a closer genetic relationship to *Glycine max* MFT (GmMFT), *Citrus unshiu* MFT (CuMFT) and *Jatropha curcas* MFT1 (JcMFT1); whereas GhMFT2 showed a closer genetic relationship to *Citrus limon* MFT (CiMFT), JcMFT2 and HbMFT1 (Fig 1B).

### Expression of *GhMFT* homoeologous genes in *G*. *hirsutum*

**Fig 2:**
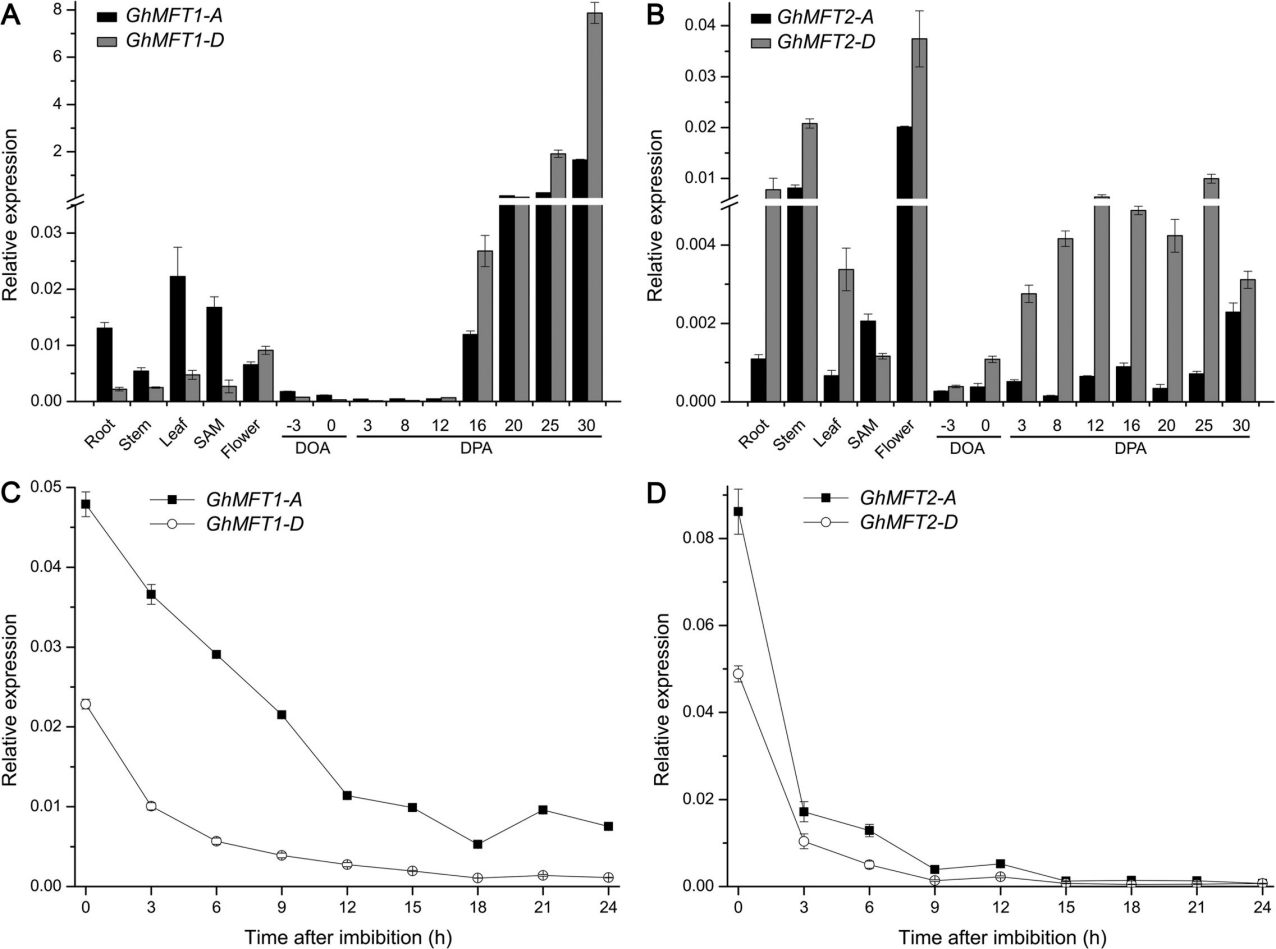
Expression patterns of *GhMFT1* and *GhMFT2* homoeologous genes. Expression patterns of *GhMFT1* (A) and *GhMFT2* (B) homoeologous genes in various tissues and at different stages of ovule development. Roots, stems, leaves and shoot apical meristems (SAM) were sampled at the third true-leaf stage, and a whole flower was collected at the fiowering stage. Ovules were sampled on -3 and 0 d of anthesis (DOA), 3, 8, 12, 16, 20, 25 and 30 d of post-anthesis (DPA). Expression patterns of *GhMFT1* (C) and *GhMFT2* (D) homoeologous genes in germinating seeds. A cotton *Ubiquitin7* (*GhUBQ7*, GenBank accession no. DQ116441) gene was used as an internal control. Data represent mean ± SEM of three independent assays.

To further understand the potential functions of *GhMFT* homoeologous genes, expression patterns of two Upland cotton homoeologous *MFT* genes were analyzed in different tissues including roots, stems, leaves, the SAM, fiowers, and at different developmental stages of ovules using qRT-PCR. The transcripts for two homoeologous *MFT* gene pairs were detected in all the investigated samples with a certain difference in their expression levels (Fig 2). The expression level of *GhMFT1-A* in roots, leaves and the SAM was higher than that of *GhMFT1-D*, whereas *GhMFT1-D* was highly expressed in ovules (Fig 2A). During ovule development, *GhMFT1* homoeologous genes had similar expression patterns. Their expression levels were significantly increased at the ovule of 16 DPA, and were continuously upregulated and peaked at ovule of 30 DPA. The expression of *GhMFT1-D* was higher than that of *GhMFT1-A* during ovule development. *GhMFT2* homoeologous genes were also expressed in roots, stems, leaves, the SAM and flower, with the highest expression level in flower (Fig 2B). Furthermore, the expression level of *GhMFT2*-*D* was significantly higher than that of *GhMFT2-A* in each tissue except in SAM. During ovule development, the expression level of *GhMFT2-D* was significantly higher than that of *GhMFT2-A*. *GhMFT2-D* was upregulated during ovule development from -3 DOA to 12 DPA and peaked at 12 DPA, and then downregulated. However, the expression level of *GhMFT2-D* in the ovule of 25 DPA reached the highest point during the of ovule development. Our results revealed that the expression patterns of *GhMFT1* and *GhMFT2* homoeologous genes were slightly different, suggesting that they may play differential roles in regulating ovule development.

To explore whether *GhMFT* homologous genes played roles in cotton seed germination, we tested their expression levels during seed imbibition. The results revealed that expressions of both *GhMFT* homologous genes decreased during cotton seed imbibition. The mRNA levels of *GhMFT1* homoeologous genes gradually decreased during 0–12 h of imbibition, after that they maintained at relatively stable levels (Fig 2C). However, expressions of *GhMFT2* homoeologous genes decreased immediately in the early stages of seed germination (0–3 h), and downregulated gradually during 3–9 h, and kept at a relatively stable level after 9 h (Fig 2D). Furthermore, our results showed that the expression levels of the A subgenome were higher than that of D subgenome, implying that *GhMFT1*-*A* and *GhMFT2-A* may play major roles during seed germination.

**Fig 3:**
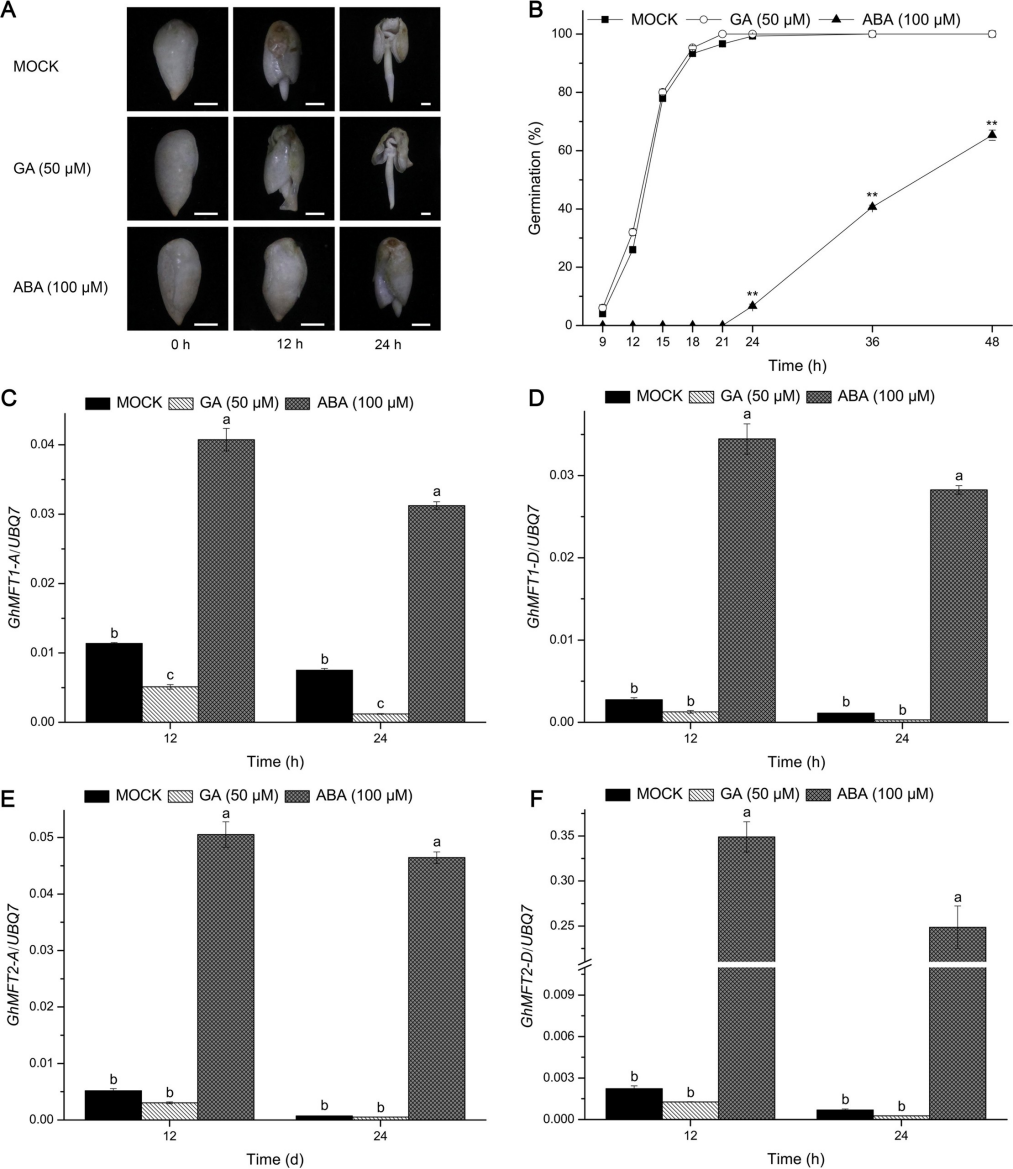
Expression patterns of *GhMFT1* and *GhMFT2* homoeologous genes in germinating seeds treated with exogenous GA and ABA. (A) Growth of germinating seeds of cotton with or without GA/ABA. The asterisks represent significant differences compared with controls (*P* < 0.01, Student’s *t*-tests). (B) Germination percentages of cotton seeds treated with 50 μM GA and 100 μM ABA. Expression of *GhMFT1-A* (C) and *GhMFT1-D* (D) in cotton seeds at 12 h and 24 h of treatment with 50 μM GA and 100 μM ABA. Expression of *GhMFT2-A* (E) and *GhMFT2-D* (F) in cotton seeds at 12 h and 24 h of treatment with 50 μM GA and 100 μM ABA. A cotton *Ubiquitin7* gene (*GhUBQ7*, GenBank accession no. DQ116441) was used as an internal control. Data represent mean ± SEM of three independent assays. Different lowercase letters represent statistically significant differences as determined by one-way ANOVA (*P* < 0.05, Duncan’s multiple range tests). Scale bar, 3 mm.

Six putative ABA-responsive elements (ABREs) were found to be located ~1850 bp upstream of the start codon of *AtMFT* (S3 Fig). These ABREs are necessary for ABA to regulate *AtMFT* expression, and *AtMFT* acts as a negative regulator in response of ABA [5]. Several putative ABREs were also identified in the approximate 1.8 kb upstream of the initiation codon of two *GhMFT* homoeologous genes through promoter analysis. There is a single ABRE located ~153 bp upstream of the translational start site of *GhMFT1* homoeologous genes, whereas a separate cluster of three ABREs located ~142 bp upstream of the start codon of *GhMFT2* homoeologous genes (S3 Fig). The presence of these ABREs in the promoters of *GhMFT* homoeologous genes hints that ABA might regulate their expressions. We next investigated whether the expression levels of *GhMFT* homoeologous genes were influenced by GA or ABA. To answer this question, we detected changes in their transcription levels in response to exogenous GA and ABA treatments at 12 and 24 h of imbibition by qRT-PCR. As is shown in Fig 3B, the control seeds and seeds treated by GA started to germinate after 9 h of imbibition, and GA treatment slightly promoted seed germination, whereas this process was obviously inhibited by ABA. These results indicated that GA and ABA treatments have certain effects on cotton seed germination. In this process, the expression levels of *GhMFT* homoeologous genes were significantly increased in response to ABA treatments (Fig 3C–3F). However, their expressions showed different expression patterns in response to GA treatment. The expression of *GhMFT1-A* was notably decreased in response to GA treatment (Fig 3C). However, there were no significant changes in the expression levels of *GhMFT1-D* and *GhMFT2* homoeologous genes under GA treatment (Fig 3D–3F). In summary, these results indicated that *GhMFT* homoeologous genes may be involved in the control of seed germination of cotton in response to ABA.

### Overexpression of *GhMFT1* and *GhMFT2* do not affect the flowering time in transgenic *Arabidopsis*

To investigate whether *GhMFT* homologous genes were involved in the control of flowering time and plant architecture, we generated homologous transgenic *Arabidopsis* plants in wild type or *mft-2* background. Eight independent *35S*:*GhMFT1* homozygotes and 10 independent *35S*:*GhMFT2* homozygotes in wild type background were generated; and eight independent *35S*:*GhMFT1* homozygotes and six independent *35S*:*GhMFT2* in *mft-2* background were also generated. S4 Fig displayed the expression levels of *GhMFT* homologous genes in the representative transgenic *Arabidopsis* plants. Through phenotypic observation, we found that overexpression of *GhMFT1* and *GhMFT2* did not cause any obvious morphological changes in transgenic *Arabidopsis* under LD conditions (Panels A and B in S5 Fig; Panels A and B in S6 Fig). In addition, we found that there were no significant differences in flowering times and rosette leaf numbers among all lines not only in wild type (Panels C and E in S5 Fig; Panels C and E in S6 Fig) but also in *mft-2* (Panels D and F in S5 Fig; Panels D and F in S6 Fig) background, suggesting that *GhMFT1* and *GhMFT2* did not regulate flowering time in *Arabidopsis*.

### Overexpression of *GhMFT1* and *GhMFT2* inhibits seed germination in transgenic *Arabidopsis*

**Fig 4:**
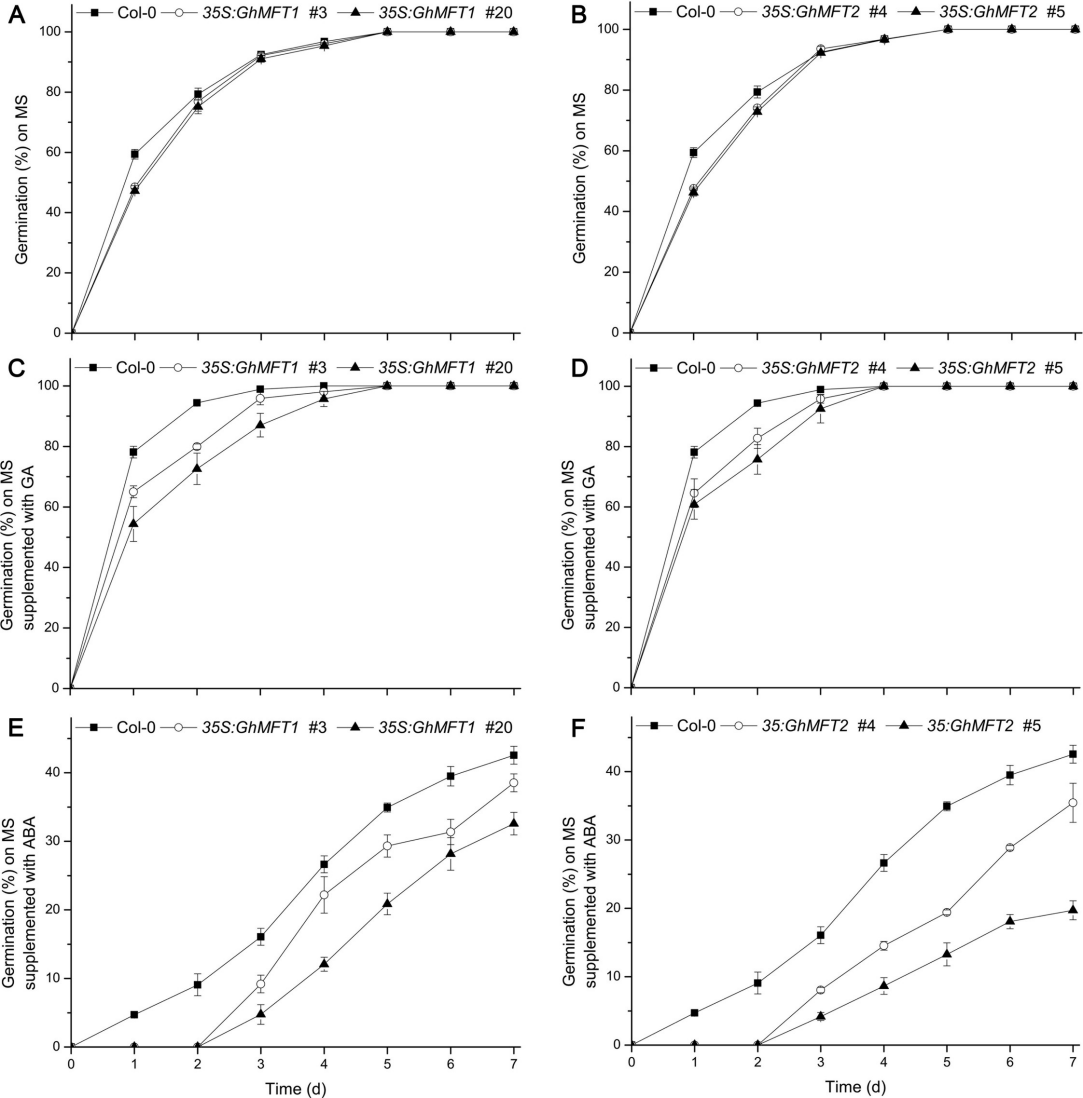
Germination phenotype of *35S*:*GhMFT1* and *35S*:*GhMFT2* transgenic *Arabidopsis* in wild type background. Germination phenotype of two representative *35S*:*GhMFT1* lines (A) and *35S*:*GhMFT2* lines (B) on 1/2 MS medium, respectively. Germination phenotype of *35S*:*GhMFT1* lines (C) and *35S*:*GhMFT2* (D) lines in response to 5 μM GA. Germination phenotype of *35S*:*GhMFT1* lines (E) and *35S*:*GhMFT2* lines (F) in response to 10 μM ABA. Data represent mean ± SEM of three independent assays.

Considering the expression patterns of *GhMFT1* and *GhMFT2* during seed germination, we guessed that they may play important roles in the regulation of seed germination. To confirm this hypothesis, the seed germination rates of different *35S*:*GhMFT1* and *35S*:*GhMFT2* transgenic lines were compared with those of wild type and *mft-2*, respectively. The result revealed that the germination rate of *35S*:*GhMFT1* transgenic seeds in wild type background was much lower than that of wild type at the early stage of seed germination (1 d after imbibition) (Fig 4A), and the germination rate of *35S*:*GhMFT2* transgenic seeds remained much lower than that of wild type on the second day (Fig 4B). Similarly, the *35S*:*GhMFT1* and *35S*:*GhMFT2* transgenic seeds in *mft-2* background also had lower germination rates than those of *mft-2* within 3 d of imbibition (Panels A and B in S7 Fig). In addition, we noticed a correlation between the expression levels of *GhMFT1* and *GhMFT2* and the germination rates of transgenic lines not only in wild type but also in *mft-2* background (Fig 4A and 4B; S4 Fig; Panels A and B in S7 Fig).

To investigate whether the seed germination of *35S*:*GhMFT1* and *35S*:*GhMFT2* was influenced by GA and ABA treatments, we next analyzed the germination rates of all transgenic plants and controls under two treatments, respectively. The results revealed that GA treatment boosted germination of all plants, but the germination rate of wild-type seeds was higher than those of *35S*:*GhMFT1* and *35S*:*GhMFT2* transgenic plants in wild type or *mft-2* background (Fig 4C and 4D; Panels C and D in S7 Fig). The germination rates of the *35S*:*GhMFT1/2 mft-2* transgenic lines were increased when exogenous GA was applied, but their germination rates could not reach the same level as that of *mft-2* in normal conditions (Panels C and D in S7 Fig). However, the seed germination rate of *mft-2* showed little difference in response to exogenous GA compared with that of wild type. Under ABA treatment, *35S*:*GhMFT1* and *35S*:*GhMFT2* transgenic plants in the wild type background began to germinate from the third day, and the germination rates were significantly lower than that of wild type (Fig 4E and 4F), whereas the transgenic plants seeds in the *mft-2* background began to germinate from the fifth day, and the germination rates were significantly lower than that of *mft-2* (Panels E and F in S7 Fig). Moreover, the germination rates of wild type and *mft-2* were lower under ABA treatment than that in normal conditions. These results revealed that ectopic expression of *GhMFT1* and *GhMFT2* repressed seed germination of *Arabidopsis* at the early stage.

### *GhMFT1* and *GhMFT2* influences the expression of ABA and GA signaling pathway genes in transgenic *Arabidopsis*

**Fig 5:**
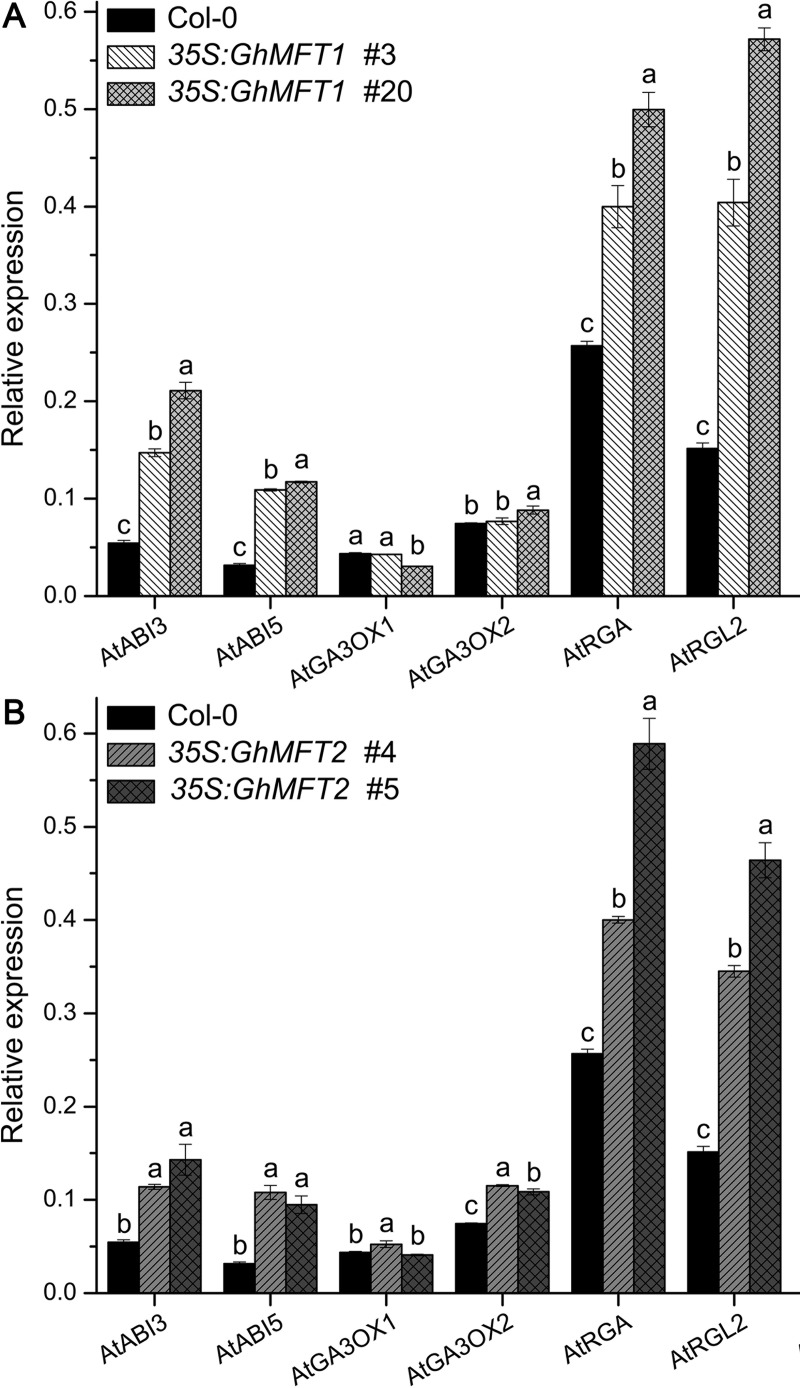
Expression of germination-related genes in wild-type, *35S*:*GhMFT1* and *35S*:*GhMFT2 Arabidopsis* seeds. All germinating seeds collected after 24 h of stratification were used for qRT-PCR. (A) Expression of *AtABI3*, *AtABI5*, *AtGA3OX1*, *AtGA3OX2*, *AtRGA* and *AtRGL2* in wild type and two representative *35S*:*GhMFT1* lines. (B) Expression of *AtABI3*, *AtABI5*, *AtGA3OX1*, *AtGA3OX2*, *AtRGA* and *AtRGL2* in wild type and two representative *35S*:*GhMFT2* lines. The *Arabidopsis Actin2* (AT3G18780) was used as an internal control. Data represent mean ± SEM of three independent assays. Different lowercase letters represent statistically significant differences as determined by one-way ANOVA (*P* < 0.05, Duncan’s multiple range tests).

To explore the possible mechanisms of *GhMFT1* and *GhMFT2* in repressing seed germination, we further analyzed the expression levels of germination-related genes, including ABA and GA signaling pathway genes among wild type, *35S*:*GhMFT1* and *35S*:*GhMFT2* transgenic plants. *AtABI3* and *AtABI5* showed higher expression levels in all the *35S*:*GhMFT1* and *35S*:*GhMFT2* transgenic plants than those of wild-type seeds (Fig 5), suggesting that *GhMFT1* and *GhMFT2* may be involved in the ABA accumulation in transgenic *Arabidopsis* seeds and control seed germination by upregulating *AtABI3* and *AtABI5* expression.

GA3OX1 and GA3OX2 are the key rate-limiting enzymes in GA synthesis pathway in *Arabidopsis*, whereas RGA and RGL2 belong to DELLA family members involving in repressing seed germination [14, 22, 25]. qRT-PCR results showed there were slightly differences in the expression of *AtGA3OX1* and *AtGA3OX2* among all detected plants (Fig 5), implying that *GhMFT1* and *GhMFT2* may affect GA synthesis. However, the expression levels of *AtRGA* and *AtRGL2* were significantly upregulated in all transgenic plants, suggesting that more DELLA proteins may be accumulated in all overexpressing *GhMFT1* and *GhMFT2* lines, resulting in inhibiting seed germination.

### Both GhMFT1 and GhMFT2 interact with GhFD protein

**Fig 6:**
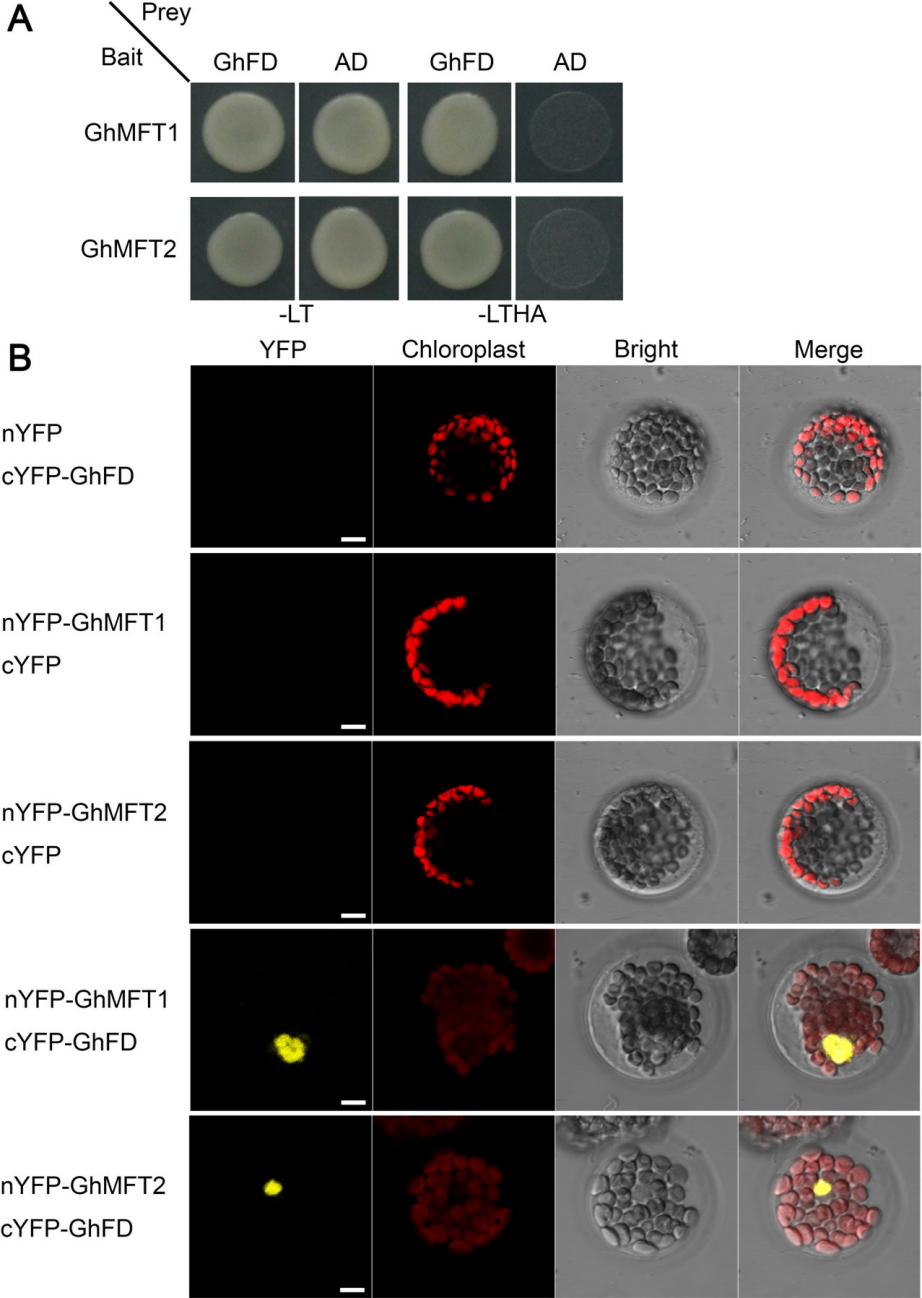
Interaction of GhMFT1 and GhMFT2 with GhFD *in vivo*. (A) Yeast two-hybrid assay of protein interaction. Transformed yeast cells harboring GhFD fused to activation domain (AD), and GhMFT1, GhMFT2 fused to binding domain (BD) were grown on selection media without Leu, Trp, His and Ade (-LTHA) indicating positive interactions. (B) Bimolecular fluorescence complementation analysis of protein interactions both GhMFT1 and GhMFT2 with GhFD in *Arabidopsis* protoplast cells. YFP, YFP fluorescence; Chlorophyll represents chloroplast auto fluorescence; Bright, bright field image; Merge represents merge of the former three images. Scale bar, 5 μm.

In the SAM of *Arabidopsis* and rice, FT interacts with florigen receptor 14-3-3 proteins and a bZIP transcription factor FD to induce transcription of floral meristem identity genes [3, 78,79]. Recent studies reveal that cotton FT homolog GhFT and TFL1/SP homolog GhSP interact with a cotton bZIP transcription factor GhFD [64, 68, 69, 71]. To analyze the subcellular mechanisms of cotton MFT homologs and their interaction with FD, we generated constructs containing GhMFT1 and GhMFT2 C-terminal fusions with GFP under control of the *CaMV 35S* promoter, which were transiently expressed in leaf epidermal cells of *N*. *benthamiana*, subsequently, the fluorescence was observed by CLSM510. As is shown in S8 Fig, green fiuorescence of the GhMFT1-GFP and GhMFT2-GFP fusion proteins was found in the peripheral cytoplasm (surrounding the vacuole) as well as in the nucleus, which was similar to that observed in cells expressing GFP alone, showing that the subcellular localization of GhMFT1 and GhMFT2 appears to be similar to that of GhFT1 [66, 67] and GhSP [68]. Yeast two-hybrid analysis confirmed that both GhMFT1 and GhMFT2 interacted with GhFD (Fig 6A). Furthermore, we observed strong fluorescence in the nucleus of *Arabidopsis* protoplasts that co-expressed GhFD with GhMFT1 or GhMFT2 by BiFC (Fig 6B), which further confirmed these results of proteins interaction.

## Discussion

### *GhMFT*-like genes may have conserved function in cotton

Identification and evolution analysis of the PEBP family genes in a wide range of land plants showed that MFT is the ancestor of FT/TFL1-like [26, 27, 29]. A number of studies have shown that *FT*/*TFL1*-like genes function in regulating flowering time and shoot meristem activity, thus contributing to each plant’s architecture [31–36, 46, 63, 68, 71]. The exact biology functions of PEBP genes in cotton are not well understood, especially *MFT*-like clade. Sequencing of cotton genome has expanded our exploration of the PEBP family. Two *MFT* homoeologous gene pairs, *GhMFT1* and *GhMFT2*, were identified according to cotton genome sequences dataset in the study [58, 60]. In *Arabidopsis*, loss-of-function mutant in *MFT* does not show defects in flowering time, and its overexpression slightly accelerates flowering, suggesting that it functions as a weak inducer [37]. Similarly, in the present study, we found that the flowering time and the number of rosette leaves in the overexpressing *GhMFT1* and *GhMFT2* plants were not significantly different from those of wild type and *mft-2* (S5 and S6 Figs). Similar results have been found in various plant species, such as *Populus nigra* [41], *Populus* [42], *Picea abies* [27], *Symplocarpus renifolius* [43], *Glycine max* [44], *Actinidia chinensis* [45], and *Citrus latifolia* [46]. A comparison of protein sequences has revealed that the key amino acid residue W in cotton MFT homologs replaces Y in FT or H in TFL1 (Fig 1A), respectively, wherein this residue lies at the entrance to a ligand-pocket and plays a crucial role in determining protein specificity as flowering inducer or repressor [32, 33], suggesting that two cotton *MFT* homologs may not be involved in regulating floral transition. Furthermore, GhMFT1 and GhMFT2 have a conserved P residue near the C terminus (Fig 1A), which is absent in FT-like or TFL1-like proteins [26]. Phylogenetic analysis revealed that GhMFT1 and GhMFT2 belong to the MFT-like subfamily (Fig 1B). These results suggest that MFT homologs may have conserved function during cotton development, whereas their functions are different from those of FT and TFL1.

### Two *GhMFT* homoeologous genes are differentially expressed during cotton ovule development and seed germination

Like *AtMFT* expression, most of the identified *MFT-*like genes in various plant species display a high expression level in seeds, suggesting a highly conserved function for *MFT*-like genes is involved in seed development, seed dormancy and seed germination across the plant kingdom, such as *TaMFT* [47], *JcMFT1* and *JcMFT2* [80, 81], and *GmMFT* [44]. In this study, we discovered that the expression levels of *GhMFT1* homoeologous genes in ovules were higher than those in other tissues, and increased significantly at the later stage of ovule development (Fig 2A). Compared with *GhMFT2-A*, *GhMFT2-D* is highly expressed during ovule development (Fig 2B), suggesting that *GhMFT2*-*D* played a major role in ovule development. However, the expression levels of *GhMFT1* homoeologs were higher than those of *GhMFT2* homoeologous genes during ovule development (Fig 2) and their expression patterns were very similar to that of *MFT* in *Arabidopsis* and soybean [5, 44]. These results imply that two cotton *MFT* homologs may be involved in the regulation of ovule development redundantly or differently.

During seed germination, the expression levels of *GhMFT1* and *GhMFT2* decreased significantly, which were similar to that of *GmMFT* [44]. The expression levels of *GhMFT1* homoeologous genes were gradually decreased at the early stage of seed germination (Fig 2C), whereas *GhMFT2* homoeologous genes were sharply reduced (Fig 2D). We found that *GhMFT1-A* expression was higher than *GhMFT1-D* during seed germination and *GhMFT2-A* was also higher than *GhMFT2-D* at the early stage of seed germination, suggesting that A subgenome of *GhMFT* plays more important roles in the regulation of seed germination. To sum up, the increase of expression during ovule development and the decrease of expression during seed germination suggest that *GhMFT* homologs may be involved in the regulation of ovule development and seed germination. The differential expression patterns of *GhMFT* homologous genes support this view that the homoeologous gene pairs from A subgenome and D subgenome complementarily contribute to Upland cotton agronomic traits [60].

### Expression of cotton *MFT* homologs is mediated by ABA and GA

Seed germination is regulated by two major antagonistic plant hormones, ABA and GA [9]. Expression of *AtMFT* is upregulated throughout seed germination when seeds are treated with exogenous ABA [5]. There were several ABREs in upstream of the start codon of *AtMFT*, which regulated expression of *AtMFT* (S3 Fig). We found that there were also several ABREs in upstream of the promoters of *GhMFT* homoeologous genes, imply that ABA might regulate their expressions, just like *AtMFT* in *Arabidopsis*. In this study, the time-point expression patterns of *GhMFT1* and *GhMFT2* homoeologous genes during seed imbibition demonstrated that they were changed in response to ABA and GA (Fig 3). We found that the expressions of *MFT* homoeologous gene pairs were remarkably enhanced in response to ABA, which were similar to those of *GmMFT* and *Dimocarpus longan MFT* (*DlMFT*) treated by 10 μM ABA [44, 53]. *GhMFT1*-*A* expression was markedly downregulated with exogenously applied GA, which is similar to *AtMFT* treated by 10 μM GA [5], implying that cotton *MFT* homologs play roles in seed germination through mediating the interaction between ABA and GA signals.

### *GhMFT* homologs redundantly and differentially regulate seed germination

In *Arabidopsis*, *mft-2* mutant does not exhibit observable defects compared with wild type under normal conditions [37]. Seeds of *mft-2* are hypersensitive to exogenous ABA and exhibit lower germination rate than that of wild type [5]. The germination rate of *mft-2* is not significantly different from that of wild type in normal conditions, but its germination rate is much lower than that of wild type in the presence of exogenous ABA indicating that seeds of *mft-2* are hypersensitive to exogenous ABA and exhibit lower germination rate than that of wild type [5]. However, seeds of *mft-2* are more insensitive to GA than that of wild type and the seed germination rate shows little difference in response to exogenous GA compared with that of wild type due to the regulation of intrinsic ABI3, ABI5 and DELLA proteins in seed germination [5]. Ectopic overexpression of soybean *GmMFT* in *Arabidopsis* does not affect flowering time, but inhibits the seed germination at the early stage [44]. Furthermore, *TaMFT* repressed seed germination [47]. Under far-red light, *AtMFT* represses seed germination by modulating ABA and GA responses in *A*. *thaliana* [50]. In this study, we found that ectopic overexpression of *GhMFT* homologs in *A*. *thaliana* also significantly inhibited seed germination by generating *35S*:*GhMFT1* and *35S*:*GhMFT2* transgenic lines, respectively (Fig 4 and S7 Fig). The germination rates of the *35S*:*GhMFT1/2 mft-2* transgenic lines could not reach the same level as that of *mft-2* in normal conditions when exogenous GA was applied (Panels C and D in S7 Fig), indicating that the phenotypes of *mft-2* on seed germination are not fully complemented by *GhMFT1/2* overexpression. Moreover, overexpression of cotton *MFT* homologs could not rescue the ABA-sensitive phenotype of *mft-2*, and the germination rates of the transgenic lines in *mft-2* background were much lower than *mft-2* with ABA (Panels E and F in S7 Fig), implying that *GhMFT* homologs aggravated the sensitivity of *mft-2* to ABA. To sum up, we guess that *GhMFT1* and *GhMFT2* may act as negative regulators of seed germination as *AtMFT* in far-red light [50], *GmMFT* [44] and *TaMFT* [47] do.

In *Arabidopsis* germinating seeds, *AtABI5* upregulates *MFT* expression but *AtABI3* suppresses *MFT* expression, whereas DELLA proteins RGA and RGL2 also upregulate *MFT* expression [5]. Under far-red light conditions, the accumulation of the transcription factor PHYOCHROME-INTERACTING-FACTOR1 (PIF1) promoted the expression of genes encoding ABI5 and DELLA growth-repressor proteins. *MFT* gene expression was promoted by far-red light through the PIF1/SOMNUS (SOM)/ABI5/DELLA pathway, and then *MFT* repressed seed germination [50]. In this study, we found that the expression levels of *AtABI3* and *AtABI5* were notably elevated in *GhMFT1* and *GhMFT2* transgenic lines (Fig 5). Moreover, the expression levels of *AtRGA* and *AtRGL2* were also found to be remarkably elevated in all transgenic plants. Based on these results, we speculate that *GhMFT1* and *GhMFT2* may inhibit seed germination by increasing the concentration of ABA in germinating seeds. However, the GA synthetic pathway genes, *AtGA3OX1* and *AtGA3OX2* showed differential expression patterns in different transgenic lines of *35S*:*GhMFT1* and *35S*:*GhMFT2*, suggesting that *GhMFT1* and *GhMFT2* regulate seed germination differentially in GA pathway. This hypothesis needs further confirmation.

Recent studies showed that cotton FT and TFL1/SP-like proteins compete for interaction with GhFD, involving in the regulation of plant architecture and flower morphology [64, 68, 69, 71]. As Prewitt et al. [71] reported that cotton GhMFT1 can interact with *G*. *raimondii* FD (GrFD) in yeast cells, we also found that both GhMFT1 and GhMFT2 can interact with GhFD not only in yeast cells but also in *Arabidopsis* protoplast cells (Fig 6). Since GhMFT1 and GhMFT2 are located in the cytoplasm and nucleus (S8 Fig), we hypothesize that two MFT-FD proteins may act as transcription co-regulators that regulate the expression of genes related ABA pathway, but the roles for these complexes in ovule development and seed germination of cotton needs further study.

## Conclusions

Genome-wide analysis identified two *MFT* homoeologous gene pairs in *G*. *hirsutum*, namely, *GhMFT1-A/D* and *GhMFT2-A/D*. Expression of *GhMFT1* and *GhMFT2* homoeologs remarkably increased during ovule development but quickly decreased during seed germination. Their expression levels were significantly affected by ABA. Ectopic overexpression of *GhMFT1* and *GhMFT2* in *Arabidopsis* repressed seed germination at the early stage. Moreover, the expression levels of *AtABI3*, *AtABI5*, *AtRGA* and *AtRGL2* were obviously upregulated in transgenic *Arabidopsis* seeds. Both GhMFT1 and GhMFT2 interact with a bZIP transcription factor GhFD. Taken together, we speculate that *GhMFT1* and *GhMFT2* may act redundantly in the regulation of seed germination.

## Supporting information

S1 Fig Partial nucleotide sequences comparison of *GhMFT*-like genes in cotton. (A) Alignment of partial nucleotide sequences of cotton *MFT1* homologs among A subgenome and its progenitors. (B) Alignment of partial nucleotide sequences of cotton *MFT2* homologs among D genome and its progenitors. qRT-PCR primer locations were marked with different colors. Left and right black arrows indicated the locations of forward and reverse primers, respectively. Different colors indicate the differences of nucleotides between the homoeologs of A subgenome and D subgenome.
(TIF)

S2 Fig Multiple alignment and phylogenetic relationships of GoMFT proteins sequences. (A) Alignment of amino acid sequences of cotton *GoMFT* homologous proteins. Two red boxes indicate the D-P-D-x-P and m G-x-H-R motifs, respectively. Amino acid shown in red and green is the conserved amino acid Trp (W) and Pro (P) for GoMFT, respectively. The black inverted triangle indicates the position of the intron. (B) Phylogenetic relationships and gene structures MFT homologs. The bootstrap consensus tree was inferred from 1000 replicates using MEGA 6.0. Black boxes and lines indicate exons and introns, respectively.
(TIF)

S3 Fig Promoter analysis of *MFT*-like in *G. hirsutum* and *Arabidopsis*. The sequence for *AtMFT* was derived from TAIR (https://www.arabidopsis.org/) database. Putative ABREs were identified using online Mat Inspector software (http://www.genomatix.de/) and marked by red inverted triangles respectively. Upstream region and introns are represented by white boxes, while exons are indicated by black boxes.
(TIF)

S4 Fig Expression levels of *GhMFT1* and *GhMFT2* in the representative transgenic *Arabidopsis* by qRT-PCR. (A) Expression levels of *GhMFT1* in wild type, *mft-2* and four representative *35S*:*GhMFT1* lines. (B) Expression levels of *GhMFT2* in wild type, *mft-2* and four representative *35S*:*GhMFT1* lines. Data represent mean ± SEM of three independent assays.
(TIF)

S5 Fig Phenotype analysis of transgenic *Arabidopsis* lines that ectopically expressing *GhMFT1*. (A) Representative phenotypes of 29 d wild-type *Arabidopsis* and two representative *35S*:*GhMFT1* transgenic lines grown in phytotron under LD conditions, respectively; (B) Representative phenotypes of 29 d *mft-2* and *35S*:*GhMFT1* transgenic lines grown in phytotron under LD conditions; (C, D) Flowering time of wild type, *mft-2* and *35S*:*GhMFT1* transgenic lines grown in phtyotron under LD conditions. (E, F) Rosette leaves of wild type, *mft-2* and *35S*:*GhMFT1* transgenic lines grown in phytotron under LD conditions. Lines across the boxes denote the medians. The box represents the 25th and 75th percentile. The top and bottom whisker caps depict the maximum and minimum values, respectively. The white squares represent mean values (n = 14). NS, nonsignificant difference (*P* < 0.05, Student’s *t*-tests).
(TIF)

S6 Fig Phenotype analysis of transgenic *Arabidopsis* lines that ectopically expressing *GhMFT2*. (A) Representative phenotypes of 29 d wild-type *Arabidopsis* and two representative *35S*:*GhMFT2* transgenic lines grown in phytotron under LD conditions, respectively; (B) Representative phenotypes of 29 d *mft-2* and *35S*:*GhMFT2* transgenic lines grown in phytotron under LD conditions; (C, D) Flowering time of wild type, *mft-2* and *35S*:*GhMFT2* transgenic lines grown in phtyotron under LD conditions. (E, F) Rosette leaves of wild type, *mft-2* and *35S*:*GhMFT2* transgenic lines grown in phytotron under LD conditions. Lines across the boxes denote the medians. The box represents the 25th and 75th percentile. The top and bottom whisker caps depict the maximum and minimum values, respectively. The white squares represent mean values (n = 15). NS, nonsignificant difference (*P* < 0.05, Student’s *t*-tests).
(TIF)

S7 Fig Germination phenotype of *35S:GhMFT1* and *35S:GhMFT2* transgenic *Arabidopsis* seeds in *mft-2* background. **Germination phenotype of two representative *35S*:*GhMFT1*.** (A) and *35S*:*GhMFT2* (B) transgenic lines on 1/2 MS medium, respectively. Germination phenotype of *35S*:*GhMFT1* (C) and *35S*:*GhMFT2* (D) transgenic lines on 1/2 MS medium supplemented with 5 μM GA. (E, F) Germination phenotype of *35S*:*GhMFT1* (E) and *35S*:*GhMFT2* (F) transgenic lines on 1/2 MS medium supplemented with 10 μM ABA. Data represent mean ± SEM of three independent assays.
(TIF)

S8 Fig Nucleus and cytoplasm subcelluar locations of GhMFT1-green fiuorescent protein (GFP) and GhMFT2-GFP in *N. benthamiana*. Micrographs showing cells expressing GFP (control, upper lane), GhMFT1-GFP (middle lane) and GhMFT2-GFP (bottom lane) fusion protein, which were examined under fiuorescent-field illumination (left) to examine GFP fiuorescence, and under bright-field illumination (middle), and by confocal microscopy (right) for an overlay of bright and fiuorescent illumination. Scale bar, 20 μm.
(TIF)

S1 Table Predicted *G. arboreum*, *G. raimondii*, *G. hirsutum* and *G. barbadense* MFT proteins with genome identifiers. (XLSX)

S2 Table Plant PEBP proteins used for multiple alignment and phylogenetic analysis in this study. (XLSX)

S3 Table Primers used in this Study (Sequence 5’→3’). (XLSX)
